# Increased extracellular volume and altered mechanics are associated with left ventricular hypertrophy in hypertensive heart disease, not hypertension alone

**DOI:** 10.1186/1532-429X-16-S1-P393

**Published:** 2014-01-16

**Authors:** Sujith Kuruvilla, Rajesh Janardhanan, Yang Yang, Yasmin S Hamirani, Frederick H Epstein, Ellen C Keeley, Christopher M Kramer, Michael Salerno

**Affiliations:** 1 Cardiology, University of Virginia Medical Center, Charlottesville, Virginia, USA; 2 Radiology, University of Virginia Health System, Charlottesville, Virginia, USA; 3 Biomedical Engineering, University of Virginia Health System, Charlottesville, Virginia, USA

## Background

### Study purpose

To detect differences in extracellular volume (ECV) and systolic strain in hypertensive patients with LVH as compared to hypertensive patients without LVH and age-matched controls. Background: Increased ECV due to diffuse myocardial fibrosis in hypertensive patients may be an underlying mechanism contributing to increased cardiovascular risk. Moreover HTN patients with left ventricular hypertrophy (LVH) are at higher risk of cardiovascular morbidity and mortality when compared to HTN Non-LVH subjects. We compared levels of ECV and systolic strain between HTN LVH, HTN Non-LVH and control subjects.

## Methods

T1 mapping was performed in 21 HTN LVH (55 ± 11 years), 12 HTN Non-LVH (60 ± 14 years) and 21 control (52 ± 9) subjects on a Siemens 1.5T Avanto using 3-5 MOLLI (11 heart beats, 2 inversions, 3 recovery beats, 8 images). Patients with known coronary disease, significant valvular disease, and other causes of LVH were excluded. MOLLI sequence parameters included: echo time/repetition time/flip angle 1.1 ms/2.5 ms/35°, field of view 340 × 260, resolution 1.8 mm × 1.8 mm, thickness 8 mm. T1 was determined pre-contrast and 10,15 and 20 minutes following injection of 0.15 mmol/kg Gd-DTPA. T1 maps were generated using an in-house MATLAB program. Partition Coefficient (λ) was determined from the slope of a plot of 1/T1 of the myocardium versus 1/T1 of the blood. Volume of distribution (Vd) was calculated as λ*(1-Hematocrit). LV mass and function was assessed by SSFP cine imaging. Circumferential strain measurements were performed using cine DENSE. Values were compared between groups using one-way ANOVA.

## Results

**Figure 1:**
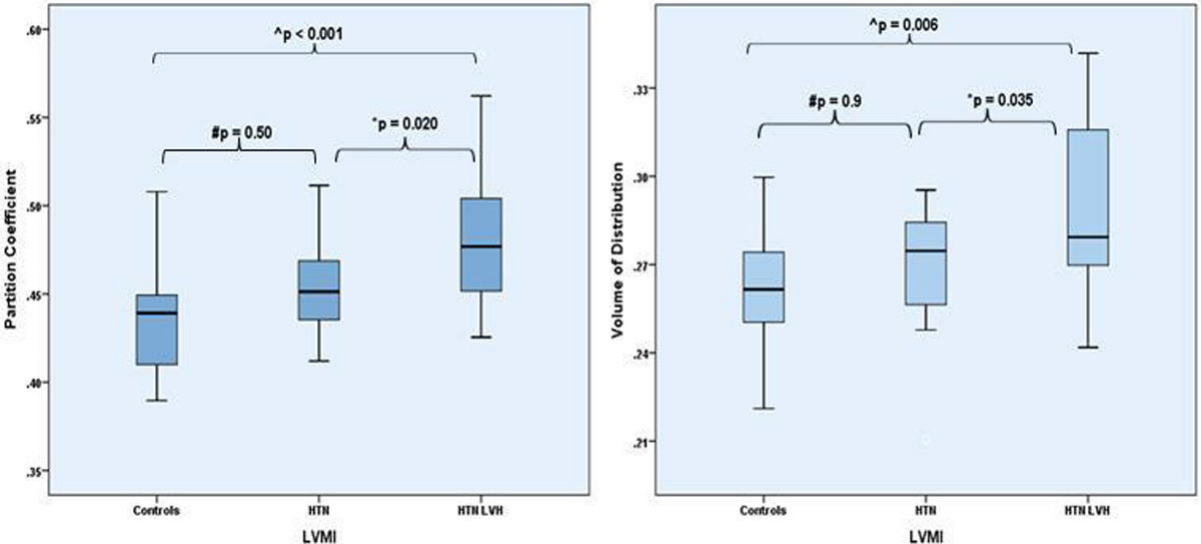
**Partition Coefficient (left) and Volume of distribution (right) among all three groups**.

HTN LVH subjects had significantly higher BP, LV mass and were on more anti-hypertensive medications when compared to HTN Non-LVH subjects and controls (Table 1). HTN LVH subjects had higher levels of ECV, as measured by λ and Vd, when compared to HTN Non-LVH and control subjects (Table 1 and Figure 1). Peak mid-wall circumferential strain was significantly reduced in HTN LVH subjects as compared to HTN Non-LVH subjects and controls (-0.12 ± 0.02 vs. -0.16 ± 0.03, p = 0.006 and -0.17 ± 0.03, p < 0.001, respectively).

**Table 1:** 

	Age-matched controls (n = 21)	Hypertensive Non-LVH (n = 12)	Hypertensive LVH (n = 21)
Sex	15 females; 6 males	6 females; 6 males	13 females; 8 males

Age (yrs)	52 ± 9	60 ± 14	55 ± 11

Systolic BP (mm Hg)	120 ± 16	136 ± 13^	156 ± 21*^

Diastolic BP (mm Hg)	69 ± 10	77 ± 12	88 ± 14^

Heart Rate	72 ± 12	72 ± 12	73 ± 16

Number of HTN meds	0	2.0 ± 1.3^	3.0 ± 1.5^

LV Mass (g)	75 ± 20	85 ± 23	147 ± 40*^

LVMI	41 ± 8	41 ± 9	71 ± 16*^

Partition Coefficient	0.43 ± 0.02	0.45 ± 0.02	0.48 ± 0.04*^

Volume of Distribution	0.26 ± 0.02	0.26 ± 0.02	0.28 ± 0.02*^

Mid-CircumferentialStrain	-0.17 ± 0.03	-0.16 ± 0.03	-0.12 ± 0.02*^

[* = p < 0.05 vs. Non-LVH groups. ^ = p < 0.05 vs. controls.]

## Conclusions

HTN LVH patients had higher ECV and associated reduction in mid-wall circumferential strain when compared to HTN Non-LVH and control subjects. Increased ECV as a surrogate for diffuse fibrosis in HTN LVH subjects may explain the increased cardiovascular morbidity and mortality seen in HTN LVH as compared to other HTN subtypes.

## Funding

AHA 10SDG2650038.

